# Low Expression of Stanniocalcin 1 (STC-1) Protein Is Associated With Poor Clinicopathologic Features of Endometrial Cancer

**DOI:** 10.3389/pore.2021.1609936

**Published:** 2021-09-28

**Authors:** Masuma Khatun, Elina Urpilainen, Anne Ahtikoski, Riikka K. Arffman, Annukka Pasanen, Ulla Puistola, Juha S. Tapanainen, Leif C. Andersson, Ralf Butzow, Mikko Loukovaara, Terhi T. Piltonen

**Affiliations:** ^1^ Department of Obstetrics and Gynaecology, PEDEGO Research Unit, Medical Research Center, Oulu University Hospital, University of Oulu, Oulu, Finland; ^2^ Department of Pathology, Oulu University Hospital, University of Oulu, Oulu, Finland; ^3^ Department of Pathology, Turku University Hospital, Turku, Finland; ^4^ Department of Pathology, University of Helsinki, Helsinki, Finland; ^5^ Department of Obstetrics and Gynaecology, Helsinki University Hospital and University of Helsinki, Helsinki, Finland

**Keywords:** stanniocalcin-1, uterine cancer, type 2 diabetes mellitus, disease-specific survival, endometrioid carcinoma, metformin

## Abstract

Stanniocalcin-1 (STC-1) is a glycoprotein hormone involved in diverse biological processes, including regulation of calcium phosphate homeostasis, cell proliferation, apoptosis, inflammation, oxidative stress responses, and cancer development. The role of STC-1 in endometrial cancer (EC) is yet to be elucidated. In this study, we investigated the protein expression pattern of STC-1 in a tissue microarray (TMA) cohort of hysterectomy specimens from 832 patients with EC. We then evaluated the prognostic value of STC-1 expression regarding the clinicopathologic features and patients survival over a period of 140 months. Our results revealed that in EC tissue samples, STC-1 is mainly localized in the endometrial epithelium, although some expression was also observed in the stroma. Decreased STC-1 expression was associated with factors relating to a worse prognosis, such as grade 3 endometrioid tumors (*p* = 0.030), deep myometrial invasion (*p* = 0.003), lymphovascular space invasion (*p* = 0.050), and large tumor size (*p* = 0.001). Moreover, STC-1 expression was decreased in tumors obtained from obese women (*p* = 0.014) and in women with diabetes mellitus type 2 (DMT2; *p* = 0.001). Interestingly, the data also showed an association between DNA mismatch repair (MMR) deficiency and weak STC-1 expression, specifically in the endometrial epithelium (*p* = 0.048). No association was observed between STC-1 expression and disease-specific survival. As STC-1 expression was particularly low in cases with obesity and DMT2 in the TMA cohort, we also evaluated the correlation between metformin use and STC-1 expression in an additional EC cohort that only included women with DMT2 (n = 111). The analysis showed no difference in STC-1 expression in either the epithelium or the stroma in women undergoing metformin therapy compared to metformin non-users. Overall, our data may suggest a favorable role for STC-1 in EC behavior; however, further studies are required to elucidate the detailed mechanism and possible applications to cancer treatment.

## Introduction

Endometrial cancer (EC) is one of the most common gynecological cancers and is the fifth most frequent malignancy among women (1). Several non-genetic risk factors, such as obesity, physical inactivity, excess estrogen effect, insulin resistance, adiposity, and diabetes mellitus type 2 (DMT2), have been documented as contributors in EC pathogenesis (2, 3). However, EC can also be caused by Lynch syndrome, an inherited condition of deficient DNA mismatch repair (MMR) that disturbs genome integrity (4, 5).

Mammalian stanniocalcin 1 (STC-1) is a 56 kDa homodimeric glycoprotein hormone. It plays a diverse role in many physiological and pathological processes such as calcium and phosphate homeostasis, organogenesis, angiogenesis, cellular metabolism, differentiation, implantation, and lactation (6-8). Zhang et al. (2000) were the first group to report about the cytoprotective activity of STC-1 in cerebral neurons against hypoxic/ischemic damage (9). Later on, in line with this, several studies considered STC-1 as a “molecular guard” because it serves as a pro-survival factor protecting against hypoxic, hypercalcaemic, and ischemic damage mainly by modulating inflammatory responses and oxidative stress (10-14). In addition to the numerous cellular functions, STC-1 has also been reported to be involved in various human cancers, including breast, ovarian, and cervical cancers, by regulating cellular proliferation, invasion, and metastasis (15-19). Furthermore, growing evidence indicates that elevated expression of STC-1 is associated with a poor prognosis in various cancers such as human esophageal squamous cell carcinoma, as well as colorectal, glioma, gastric, and breast cancers (19).

Obesity, one of the leading causes of DMT2, promotes hormonal imbalance, especially by fat tissue-derived estrogen synthesis, hyperinsulinemia, and chronic inflammation; thus, obesity can be considered a predisposing factor of EC (20, 21). Although patients with DMT2 and EC share some common risk factors, including obesity and an inactive lifestyle, DMT2 itself is found to be an independent risk factor for EC (22, 23). Metformin is considered the first-line therapy for DMT2 patients. Metformin decreases the hepatic glucose output, enhances peripheral tissue insulin sensitivity, reduces circulating insulin levels, and increases glucagon-like peptide-1 secretion (24, 25). In addition, metformin has a variety of effects on the endometrium by inhibiting endometrial cell proliferation under diabetic and estrogen-induced circumstances (26, 27). Notably, metformin has been demonstrated to inhibit proliferation and invasion of EC, supporting the association of metformin use with better EC prognosis, as seen in preclinical studies (28-30). Indeed, in a small clinical trial, metformin users presented with decreased cellular proliferation measured by ki-67 expression (31); however, in a phase III clinical study, no beneficial effect could be found of metformin on the prognosis of EC (32, 33).

Microarray data has suggested that STC-1 is expressed in endometrioid EC tissues (34); however, to date, no study exists evaluating STC-1 protein expression or its association with EC characteristics and survival. While there is limited data available regarding the involvement of STC-1 in glucose metabolism in obese mice and in patients with DMT2 (35, 36), there is no data available on STC-1 in obese patients with EC and DMT2. We investigated STC-1 protein expression patterns in EC tissues and evaluated various clinicopathologic features and outcomes of patients with EC. We also evaluated the prognostic value of STC-1 expression levels for survival over a period of 140 months. Finally, we explored the expression of STC-1 in relation to metformin use among women with DMT2 and EC.

## Materials and Methods

### Tissue Microarray of Endometrial Cancer Samples (TMA Cohort)

The tissue microarray (TMA) cohort of endometrial carcinoma samples with different histological profiles (n = 832) was collected at the Department of Obstetrics and Gynecology, Helsinki University Hospital between 2007 and 2012 from patients undergoing primary surgical treatment for EC. The detailed sample collection protocol has previously been published (37, 38).

Patient information was gathered from the hospital registers at the Helsinki University Hospital. Factors that were selected for more detailed analyses included STC-1 expression in immunohistochemistry (IHC), age, body mass index (BMI) at surgery, disease stage, histological type, grade of differentiation, depth of myometrial and lymphovascular space invasion (LVSI), tumor size, and peritoneal cytology. As myometrial invasion ≥50% (39) and tumor diameter ≥2 cm (40) have been previously reported to be indicators of poorer prognosis, we categorized EC cases into groups using these cut-off values. In addition, we categorized patients into two groups according to age (cut-off value of 65 years of age) and BMI (cut-off value of 30 kg/m^2^). All TMA EC stages were based on the current staging by the International Federation of Gynaecology and Obstetrics (FIGO) (41). The follow-up of the study subjects was carried out until February 2021. Detailed clinicopathologic characteristics of the study subjects are shown in Table 1.

**TABLE 1:** Clinicopathological data on the tissue microarray (TMA) cohort of 832 endometrial cancer (EC) patients.

Variables	Values
Age (years) [median (interquartile range)]	68 (60–75)
Body mass index (kg/m^2^) [median (interquartile range)]	27.4 (23.8–32.4)
Pelvic lymphadenectomy (no. of cases, percent)	462 (55.5%)
Pelvic-aortic lymphadenectomy (no. of cases, percent)	125 (15.0%)
FIGO 2009 Stage (no. of cases, percent)	
IA	450 (54.1%)
IB	177 (21.3%)
II	57 (6.9%)
IIIA	39 (4.7%)
IIIB	7 (0.8%)
IIIC1	47 (5.6%)
IIIC2	26 (3.1%)
IVA	0 (0%)
IVB	29 (3.5%)
Histology (no. of cases, percent)	
Endometrioid carcinoma	736 (88.5%)
Clear cell carcinoma	35 (4.2%)
Serous carcinoma	29 (3.5%)
Carcinosarcoma	17 (2.0%)
Undifferentiated carcinoma	14 (1.7%)
Neuroendocrine carcinoma	1 (0.1%)
Grade (For endometrioid only, n = 736) (no. of cases, percent)	
1	419 (50.4%)
2	206 (24.8%)
3	111 (13.3%)
Adjuvant therapy (no. of cases, percent)	
Vaginal brachytherapy	401 (48.2%)
Whole pelvic radiotherapy	121 (14.5%)
Chemotherapy	34 (4.1%)
Chemotherapy and vaginal brachytherapy	49 (5.9%)
Chemotherapy and whole pelvic radiotherapy	106 (12.7%)

FIGO, International Federation of Gynaecology and Obstetrics.

### Endometrial Cancer Samples From Women With Type 2 Diabetes (Diabetic Cohort)

The diabetic EC sample cohort consisted of women with DMT2 who were diagnosed with EC at Oulu University Hospital between 2007 and 2014 (n = 111). The data was obtained from Oulu University Hospital records and included information on the patients age, anti-diabetic medication, BMI, cancer histology, LVSI, myometrial invasion, progression, and death. All EC diagnoses were based on histology, and stages were reported in line with the latest FIGO recommendation (41). The detailed sample collection protocol has previously been published (33). The clinical background data are presented in Supplementary Table 1.

Classification of patients to metformin users and non-users was based on the anti-diabetic medication (ADM) used at the time of EC diagnosis. Patients were classified as metformin users if they had used metformin alone (n = 33), combined with any other oral ADMs (n = 22), with insulin (n = 12), or both (n = 7). On the other hand, patients were categorized as metformin non-users if they used only other forms of oral ADMs (n = 6), if they used only insulin (alone; n = 12 or combined with other oral ADMs; n = 3), or if they did not use any ADM (n = 16). To summarize, the study includes 74 metformin users and 37 non-users, and the analysis was conducted on whole block tissue differently from the TMA cohort. The distribution of anti-diabetic medication users in EC samples is presented in Supplementary Figure 1.

### Immunohistochemistry (IHC)

Representative areas of each sample were marked on the immunohistochemical slides; for the TMA slides, four duplicate 0.8 mm cores were drawn from the corresponding area of the paraffin blocks. The slides were deparaffinized with hexane. Antigen retrieval of the samples was carried out with citrate buffer in a microwave oven at 800 W for 2 min and 150 W for 10 min. Endogenous peroxidase was blocked with peroxidase-blocking solution (Dako S2023). Samples were then incubated in anti-STC-1 (Atlas Antibodies; HPA023918) primary antibody in an antibody diluent (Dako S2023) for 5 min, followed by incubation with Envision polymer (Dako K5007, Denmark) and 3, 3′ diaminobenzidine (DAB) (DAKO K5007) according to the manufacturer’s protocol. Counterstaining with hematoxylin was executed prior to adding mounting medium. Negative staining was performed on two samples using Phosphate-buffered saline (PBS) instead of primary antibody.

### Image Analysis and Scoring

**FIGURE 1:**
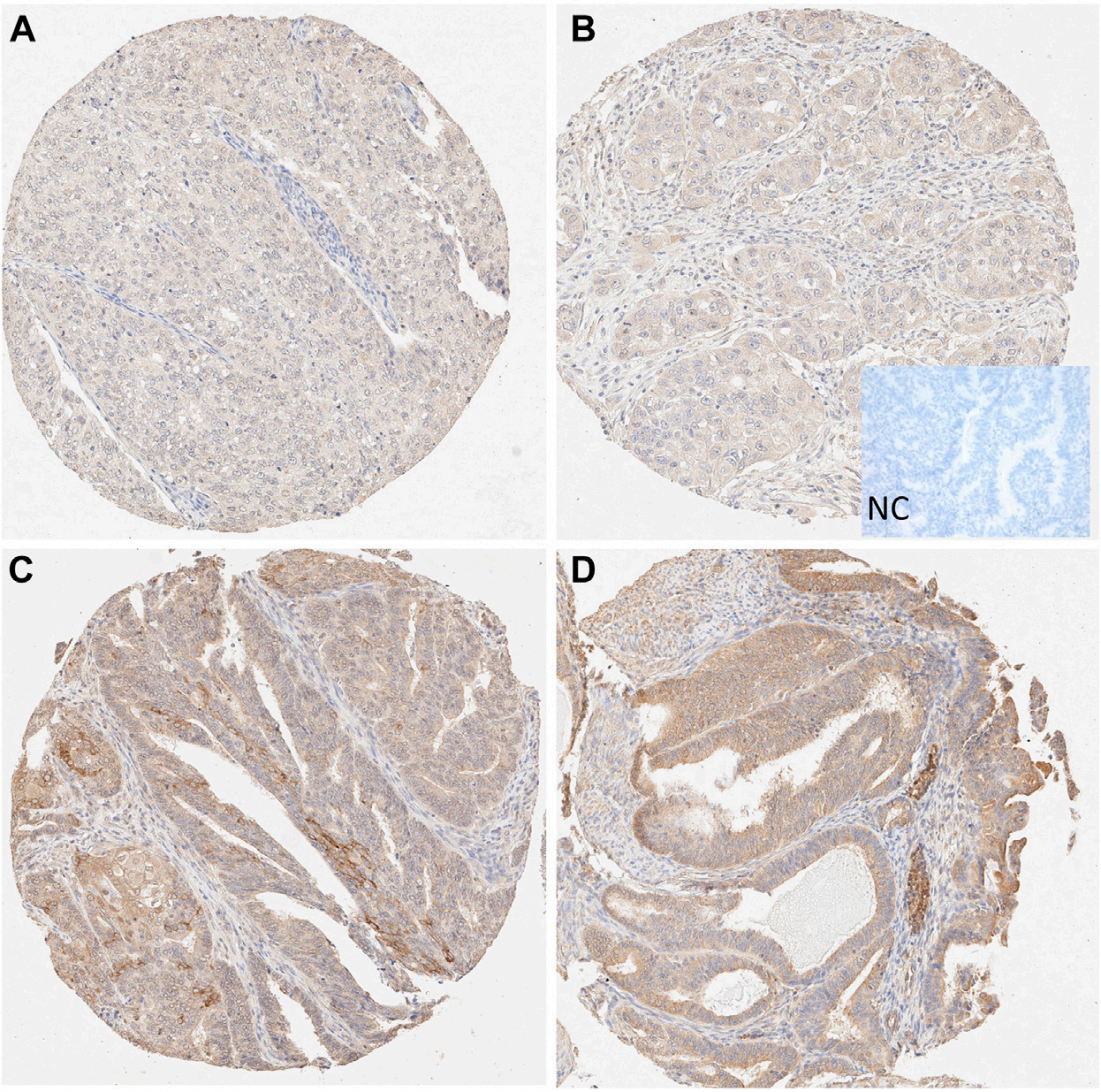
Microscopic view of STC-1 expression in the tissue microarray (TMA) samples. **(A–B)** Weak epithelial and stromal staining in grade 3 endometrioid carcinoma (10X). **(C–D)** Strong epithelial and moderate stromal staining in grade 1 endometrioid carcinoma (10X). NC, negative control.

An Aperio ImageScope (Leica Biosystems, United States) microscope was used to image the immunohistochemistry on sample slides. Semiquantitative evaluation of IHC was performed by four independent and blinded observers (MK, AA, RKA, TTP). Each sample was analyzed twice by each observer. Staining intensity was graded on a simple numeric scale as follows: score 0 = negative, 1 = faint, 2 = moderate, and 3 = intense. A consensus score of the four observers was applied to the statistical analyses. Expression levels of 0–2 were considered weak, and an expression level of 3 was considered strong for all downstream analyses in neoplastic epithelial cells for both cohorts. However, the comparison was done between scores 0–1 *versus* (*vs*) two to three in stroma due to the low proportion of scores 3 in this cell compartment. In addition, stromal staining was scored from any mesenchymal cells in the tumor background, excluding smooth muscle cells. Leucocytes or necrotic debris were not included in the scoring. Examples of the staining results from both cohorts are presented in Figure 1 and Supplementary Figure 2.

### Data Analysis

The Chi-squared test and Fisher´s exact test or Fisher-Freeman-Halton exact test were used for the comparison of categorical variables. Independent samples t-test and Mann-Whitney *U*-test were used for the comparison of continuous variables. Survivals were estimated using the Kaplan-Meier method, and differences between groups were compared with the log rank test. A statistical significance level of 0.05 was used. All analyses were performed using IBM-SPSS version 27.0 (IBM SPSS Statistics for Windows, Armonk, NY: IBM Corp).

## Results

### Endometrial Epithelium Presented With Strong Expression of STC-1 Compared to the Stroma in EC Tissue

In the TMA cohort, 99.1% (n = 825) of the EC samples stained positive for STC-1. In particular, 44.4% (n = 370) of the samples presented with intense STC-1 expression (score 3) in the epithelium samples compared to 0.3% (n = 3) in the stroma samples.

In the diabetic cohort, regardless of metformin medication, 33.3% (n = 37) of epithelium samples presented intense STC-1 expression (score 3), whereas none of the stromal samples showed the intense STC-1 expression.

### The Association Between STC-1 Expression, Clinicopathologic Features, and Outcomes of the EC Samples (TMA Cohort)

The relationships between STC-1 expression and clinicopathological features in the endometrial epithelium and stroma in EC tissues are presented in Tables 2 and 3. Weak epithelium STC-1 expression was found to be associated both with a higher BMI (*p* = 0.014) and with the presence of DMT2 (0.001). Furthermore, weaker STC-1 expression was seen in epithelium of EC with a higher grade (*p* = 0.030), LVSI (*p* = 0.050), deep myometrial invasion (*p* = 0.003), large tumour size (*p* = 0.001), and MMR deficiency (*p* = 0.048), suggesting a protective role of STC-1 in EC (Table 2). Similar to the results of the epithelium, the larger tumor size (*p* = 0.035) seemed to be associated with weaker STC-1 expression in the stroma (Table 3). However, other factors were not significantly related to STC-1 levels in the stroma.

**TABLE 2:** Comparison of variables of epithelial STC-1 expression in TMA samples of the EC cohort.

Risk variables	Weak expression (Score 0–2)	Strong expression (Score 3)	*p*-value
Age >65 years	269/462 (58.2%)	207/370 (55.9%)	0.509
**Body mass index (BMI) ≥30 kg/m^2^**	**184/462 (39.8%)**	**117/370 (31.6%)**	**0.014**
**Type 2 diabetes (DMT2)**	**105/462 (22.7%)**	**51/370 (13.8%)**	**0.001**
**Grade 3 (Endometrioid only)**	**71/401 (17.7%)**	**40/335 (11.9%)**	**0.030**
Stage II-IV	120/462 (26.0%)	85/370 (23.0%)	0.318
Non-endometrioid	61/462 (13.2%)	35/370 (9.5%)	0.093
**Myometrial invasion ≥50%**	**198/462 (42.9%)**	**121/369 (32.8%)**	**0.003**
**Lymphovascular space invasion (LVSI)**	**129/459 (28.1%)**	**80/362 (22.1%)**	**0.050**
Cervical stromal invasion	73/461 (15.8%)	46/367 (12.5%)	0.179
Positive peritoneal cytology	33/459 (7.2%)	19/364 (5.2%)	0.249
**Tumor size ≥2 cm**	**357/434 (82.3%)**	**249/346 (72.0%)**	**0.001**
**MMR deficiency**	**169/432 (39.1%)**	**114/353 (32.3%)**	**0.048**

MMR, Mismatch repair; Bold values indicates statistically significant (*p* ≤ 0.05).

**TABLE 3:** Comparison of variables of stromal STC-1 expression in TMA samples of the EC cohort.

Risk variables	Weak expression (Score 0–1) *	Strong expression (Score 2–3)	*p*-value
Age >65 years	253/461 (54.9%)	223/371 (60.1%)	0.130
Body mass index (BMI) ≥30 kg/m^2^	172/461 (37.3%)	129/371 (34.8%)	0.449
Type 2 diabetes (DMT2)	94/461 (20.4%)	62/371 (16.7%)	0.177
Stage II-IV	113/461 (24.5%)	92/371 (24.8%)	0.924
Grade 3 (Endometrioid only)	70/407 (17.2%)	41/329 (12.5%)	0.074
Non-endometrioid	54/461 (11.7%)	42/371 (11.3%)	0.860
Myometrial invasion ≥50%	178/461 (38.6%)	141/370 (38.1%)	0.882
Lymphovascular space invasion	116/454 (25.6%)	93/367 (25.3%)	0.945
Cervical stromal invasion	65/460 (14.1%)	54/367 (14.7%)	0.812
Positive peritoneal cytology	29/458 (6.3%)	23/365 (6.3%)	0.986
**Tumor size ≥2 cm**	**354/440 (80.5%)**	**252/340 (74.1%)**	**0.035**
MMR deficiency	162/431 (37.6%)	121/354 (34.2%)	0.323

MMR, Mismatch repair; Bold values indicates statistically significant (*p* ≤ 0.05), * the cut-of value is different due to the small number of score 3 samples.

The TMA cohort showed stronger epithelial STC-1 expression levels in samples taken from patients with a grade 1 EC than those taken from patients with a grade 3 EC. In detail, the STC-1 epithelial staining was cytoplasmic and mostly diffuse (Figure 1). No nuclear staining was found. The STC-1 staining in the stroma was also cytoplasmic.

**FIGURE 2:**
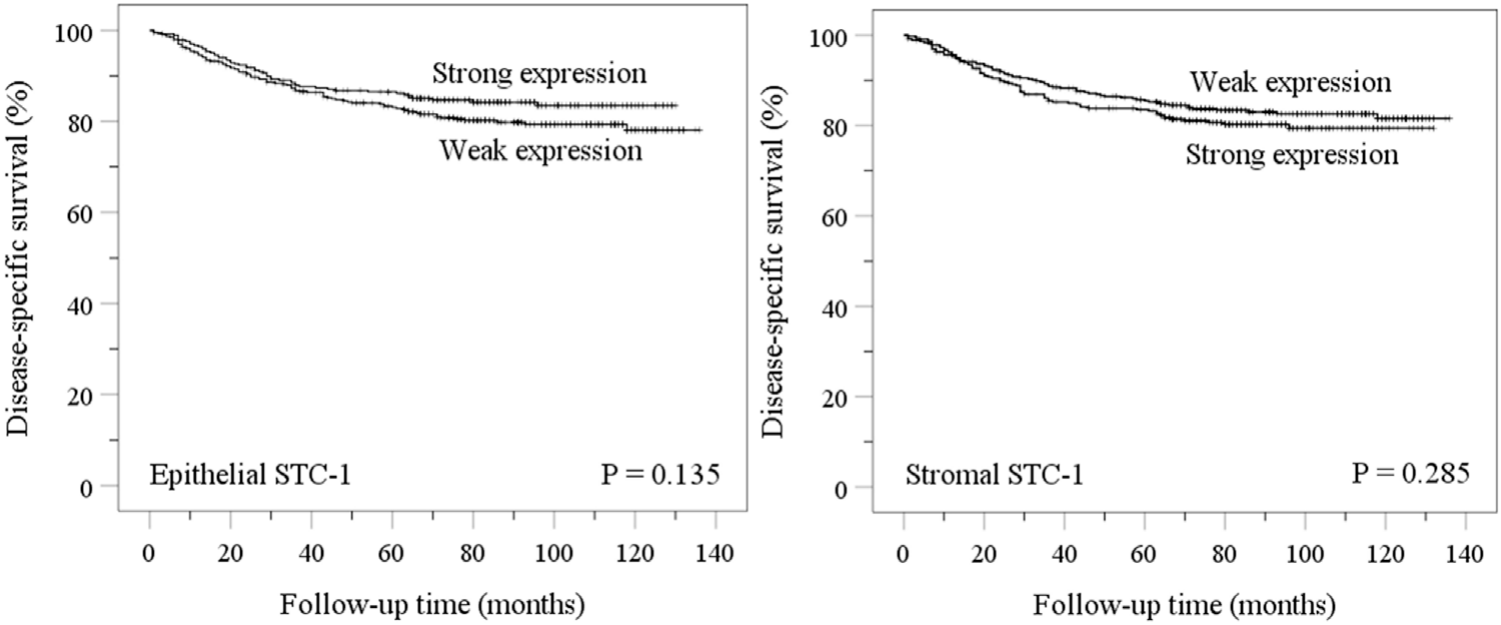
Disease-specific survival (DSS) based on the expression level of STC-1 protein in the endometrial epithelium and stroma in the TMA cohort of EC samples. Epithelium; weak expression (score 0–2), strong expression (score 3). Stroma; weak expression (score 0–1), strong expression (score 2–3).

Kaplan-Meier analyses in the TMA cohort confirmed that disease-specific survival (DSS) for EC was not associated with either endometrial epithelial (*p* = 0.135) or stromal (*p* = 0.285) STC-1 expression levels, suggesting that the prognosis is independent of STC-1 expression (Figure 2). Interestingly, in the TMA cohort, the relapse rate seemed to be different for weak epithelial STC-1 expression than for strong expression (*p* = 0.006), indicating that low STC-1 staining intensity associates with a higher rate of relapses (data not shown).

### The Association of STC-1 Expression and Clinicopathologic Features in Women With EC and DMT2 Medication (Diabetic Cohort)

There was no difference in either epithelium or stromal STC-1 expression between the metformin user and the non-user groups, indicating that metformin use is not associated with STC-1 expression (Supplementary Table 2). Metformin use was found to be linked to advanced stage (*p* = 0.011), deep myometrial invasion (*p* = 0.049), and cervical stromal invasion (*p* = 0.024). However, other tumor characteristics (type 2 histology, LVSI, positive peritoneal cytology, and tumor size) and patient characteristics (age, BMI) were similar in both groups, with and without metformin (Supplementary Table 3).

## Discussion

This is the first study investigating STC-1 protein expression in hysterectomy specimens from patients with EC. In our study, 99.15% of the samples stained positively for STC-1, and the protein was mostly located in the endometrial epithelium. These results suggest that the endometrial epithelium is the main target tissue for STC-1 and not the stroma. Subsequently, the association of STC-1 expression with various clinicopathologic features of EC suggested that weak STC-1 expression is associated with features related to poor prognosis in EC. Our study consistently showed that reduced STC-1 was slightly associated with the more aggressive MMR deficient type of EC (38, 42).

In line with recent data on STC-1 expression in the normal human endometrium, our findings indicate that the endometrial epithelium is the main target of STC-1 localization, while the stroma is not (43, 44). This also applies to hormone-dependent tumor tissues like breast and ovarian cancers underlying the role of STC-1, especially in adenocarcinoma development (17, 18). Furthermore, the localization pattern of STC-1 in the epithelium may suggest that this protein is involved in the epithelial-mesenchymal transition (EMT) process, thus, playing a crucial role in the initiation of the tumor microenvironment (45, 46). However, the role of STC-1 accumulation in the endometrial epithelium in EC warrants further studies.

In contrast to our findings, high expression of STC-1 has been shown to correlate with an advanced tumor grade in glioma and ovarian serous carcinomas, suggesting pleiotropic effects of STC-1 that depend on the type of cancer (47, 48). STC-1 has previously been shown to be involved in the advancement of aggressive metastasis and invasion by promoting cellular proliferation and reducing apoptosis in many tumor cells (17, 49-52). Furthermore, elevated STC-1 has been shown to augment cellular invasion and metastasis through the JNK/c-Jun-dependent signaling pathway in breast cancer, ovarian cancer, and gliomas (53-55). In opposition to these findings, our data showed that LVSI and myometrial invasion are associated with weak STC-1 expression. The molecular mechanism behind this contradictory finding in our case remains yet to be disclosed. Notably, our finding of the reduced expression of STC-1 being more frequent in obese and diabetic women also suggests that the substantial lack of the protein may be linked with other physiological metabolic risk factors that may not be evident in other cancer types (9, 56). Although overexpression of STC-2 (an isoform of STC-1) has been found to be responsible for metabolic dysregulation in obese mice (57), there is no data on the metabolic effects of STC-1 in obese patients with DMT2, warranting future studies in this field.

Our data showed low levels of STC-1 expression in high-grade tumors compared to low-grade ones, conflicting with the results of other studies where STC-1 has been shown to have high levels of expression associated with high-grade differentiation (19, 49, 58). For EC, this observation may be caused by the fact that high-grade (grade 3) endometrial cancers are more disorganized, whereas in benign circumstances, endometrial STC-1 is highly expressed in the normal epithelium (59, 60). Supporting our findings, the level of STC-1 expression has been found to be upregulated in terminally (post-mitotically) differentiated brain neurons (61) and fat cells (62, 63). Additionally, their findings also confirm that overexpression of STC-1 by cDNA transfection slows down the rate of proliferation, while a low level of STC-1 expression was observed in rapidly proliferating cells. Consistent with our findings, the association of reduced STC-1 expression with the worse clinicopathological outcome has also been found in cervical cancer, suggesting a role for STC-1 as a pro-apoptotic protein (16, 64). In their study, *STC-1 knock-out* mice presented with higher cell proliferation, migration, and metastasis levels. This was suggested to be accomplished *via* p65 activation of the NFκB pathway, whereas overexpression resulted in inhibition of cell proliferation and invasion by promoting cell apoptosis (16, 64). Furthermore, Yeung *et al.* (2015) reported that tumors with high STC-1 expression were significantly smaller than those with lower expression in hepatocellular carcinoma (HCC). This is also in line with our findings, confirming a pro-apoptotic effect of STC-1 *via* up-regulation of inflammatory genes that are responsible for slowing down growth and metastasis in HCC (65). Collectively, these findings confirm that weak cellular accumulation of STC-1 is associated with more aggressive cancer outcomes, which is consistent with our data.

Interestingly, despite the association of low STC-1 expression with aggressive features of EC, we were not able to show any prognostic value for STC-1 expression during a 140-months survival period. Since STC-1 acts as an anti-apoptotic survival factor, low-grade tumors with higher STC-1 expression levels might have a slower proliferation rate associated with an initially better prognosis (65, 66). However, these cases usually have extended survival with a propensity for late relapses. This trend can be observed in our Kaplan-Meier plot, where low STC-1 expression levels indicate initially worse prognosis; however, the difference seems to disappear during the extended follow-up. A similar finding was also observed in a study on breast cancer cases showing that low STC-1 expression correlated with initially aggressive tumors and early metastases, while high STC-1 expression correlated with late relapse (tumor dormancy) (67). Interestingly, the relapse rate seemed to be higher in low STC-1 cases, although more studies are needed to confirm this preliminary notion.

Finally, focusing on the link between STC-1 and metformin, we did not find any significant correlation between STC-1 expression and EC clinicopathological features in diabetic women using anti-diabetic medication. Our data does not support the idea of metformin or any other anti-diabetic medication playing a role in the modulation of STC-1 function; however, a more stringent future study is required with a larger sample size to draw a definite conclusion.

The main strength of this study was the large and well-categorized human EC sample set collected from two different study cohorts, including several clinicopathological variables. The lack of information on p53 mutation status in our study is a limitation; however, most p53-mutated endometrioid carcinomas are grade 3 tumors, which have a worse prognosis than lower grade carcinomas. However, as an exception, those tumors with both POLE (DNA polymerase epsilon) and p53 mutation have good prognosis but since POLE mutation analysis is not yet available in routine cancer diagnosis, these rare tumors have yet to be detected. In addition, a potential flaw of the TMA cohort was that even though the hysterectomy specimen was cut open while still fresh, it was fixated as a whole possibly leading to uneven tumor fixation. However, the vitality of the tumor tissue was histologically confirmed when selecting samples for the study. A limitation of the diabetic cohort study was that polypharmacy is common in patients with DMT2, so segregation of a single medication effect can be challenging, as also shown in previous retrospective clinical studies (33, 68). However, this is a pilot study paving the way for future investigations.

## Conclusion

The results indicated that the low expression of STC-1 in EC seems to be associated with factors with worse prognostic outcome, possibly implying the role of STC-1 as a protective factor against the development of EC. However, STC-1 failed to perform well as a long-term prognostic outcome measure. Our results suggest that enhancing the expression of STC-1 might be a good strategy to inhibit tumor proliferation and invasion, possibly having therapeutic implications in EC.

## Data Availability

The original contributions presented in the study are included in the article/Supplementary Material, further inquiries can be directed to the corresponding author.

## Supplementary Material

Supplementary Figure 1 Distribution of anti-diabetic medication user groups in diabetic women diagnosed with EC (diabetic cohort; n=111).

Supplementary Figure 2 Epithelial STC-1 staining intensity in the diabetic cohort. Grade 1 endometrioid carcinoma a-b: strong (score 3) staining; c-d: weak heterogeneous (score 2) staining. Grade 3 endometrioid carcinoma e-f: weak heterogeneous (score 2) staining; g-h: weak (score 1) staining. Stromal staining intensity is weak in all samples. Left column 4X, right column 20X.
