# Supplementary material for: Low Expression of Stanniocalcin 1 (STC-1) Protein Is Associated With Poor Clinicopathologic Features of Endometrial Cancer
Source: Pathol Oncol Res. 2021 Sep 28;27:1609936. doi: 10.3389/pore.2021.1609936 (PMC8505533; doi:10.3389/pore.2021.1609936)
Supplement: Supplementary file 1 [file Table1.docx]

**Supplemental Table 1: Clinicopathological data of patients with EC with and without metformin users (diabetic cohort; n=111)**

|  | **Metformin users**  **(N=74)** | **Metformin non-users (N=37)** | **P-value** |
| --- | --- | --- | --- |
|  |  |  |  |
| Age (years), Mean ± SD, Range | 70.04 ± 8.53 (51–88) | 71.73 ± 8.64 (52–88) | 0.330 |
| Body mass index (kg/m^2^), Median, Range | 34 (19–51) | 36 (22–65) | 0.463 |
| Missing | 5 (6.8%) | 5 (13.5%) |  |
| FIGO 2009 stage (no. of cases, percent) |  |  |  |
| IA | 31 (41.9%) | 24 (64.9%) | - c |
| IB | 16 (21.6%) | 7 (18.9%) |  |
| II | 8 (10.8%) | 2 (5.4%) |  |
| IIIA | 3 (4.1%) | - |  |
| IIIB | - | - |  |
| IIIC1 | 4 (5.4%) | - |  |
| IIIC2 | 3 (4.1%) | 1 (2.7%) |  |
| IVA | 1 (1.4%) | - |  |
| IVB | 6 (8.1%) | 1 (2.7%) |  |
| Unknown | 2 (2.7%) | 2 (5.4%) |  |
| Histology (no. of cases, percent) |  |  |  |
| Endometroid carcinoma | 60 (81.1%) | 32 (86.5%) | - c |
| Serous carcinoma | 9 (12.2%) | 3 (8.1%) |  |
| Mucinous carcinoma | 1 (1.4%) | - |  |
| Carcinosarcoma | - | 1 (2.7%) |  |
| Clear cell carcinoma | - | 1 (2.7%) |  |
| Undifferentiated/mixed carcinoma | 4 (5.5%) | - |  |
| Grade (For endometrioid only, n =91) |  |  |  |
| 1 | 32 (53.3%) | 22 (68.8%) | 0.349 |
| 2 | 19 (31.7%) | 8 (25.0%) |  |
| 3 | 8 (13.3 %) | 2 (6.3%) |  |
| Missing | 1 (1.7%) | - |  |
| Adjuvant treatment (no. of cases, percent) |  |  |  |
| None | 36 (48.6%) | 18 (48.6%) | 0.462 |
| WPRT | 9 (12.2%) | 4 (10.8%) |  |
| Chemotherapy | 7 (9.5%) | 2 (5.4%) |  |
| Brachytherapy | 6 (8.1%) | 6 (16.2%) |  |
| Hormonal treatment | 1 (1.4%) | 1 (2.7%) |  |
| Intracavitary radiation | 2 (2.7%) | 3 (8.1%) |  |
| Combinations | 13 (17.6%) | 3 (8.1%) |  |

FIGO, International Federation of Gynaecology and Obstetrics; c comparison not relevant

Combinations: WPRT + Brachytherapy, WPRT + Chemotherapy, Chemotherapy + Brachytherapy, Intracavitary + Hormonal treatment, Intracavitary + WPRT
