# Supplementary figures and images for: Low Expression of Stanniocalcin 1 (STC-1) Protein Is Associated With Poor Clinicopathologic Features of Endometrial Cancer
Source: Pathol Oncol Res. 2021 Sep 28;27:1609936. doi: 10.3389/pore.2021.1609936 (PMC8505533; doi:10.3389/pore.2021.1609936)

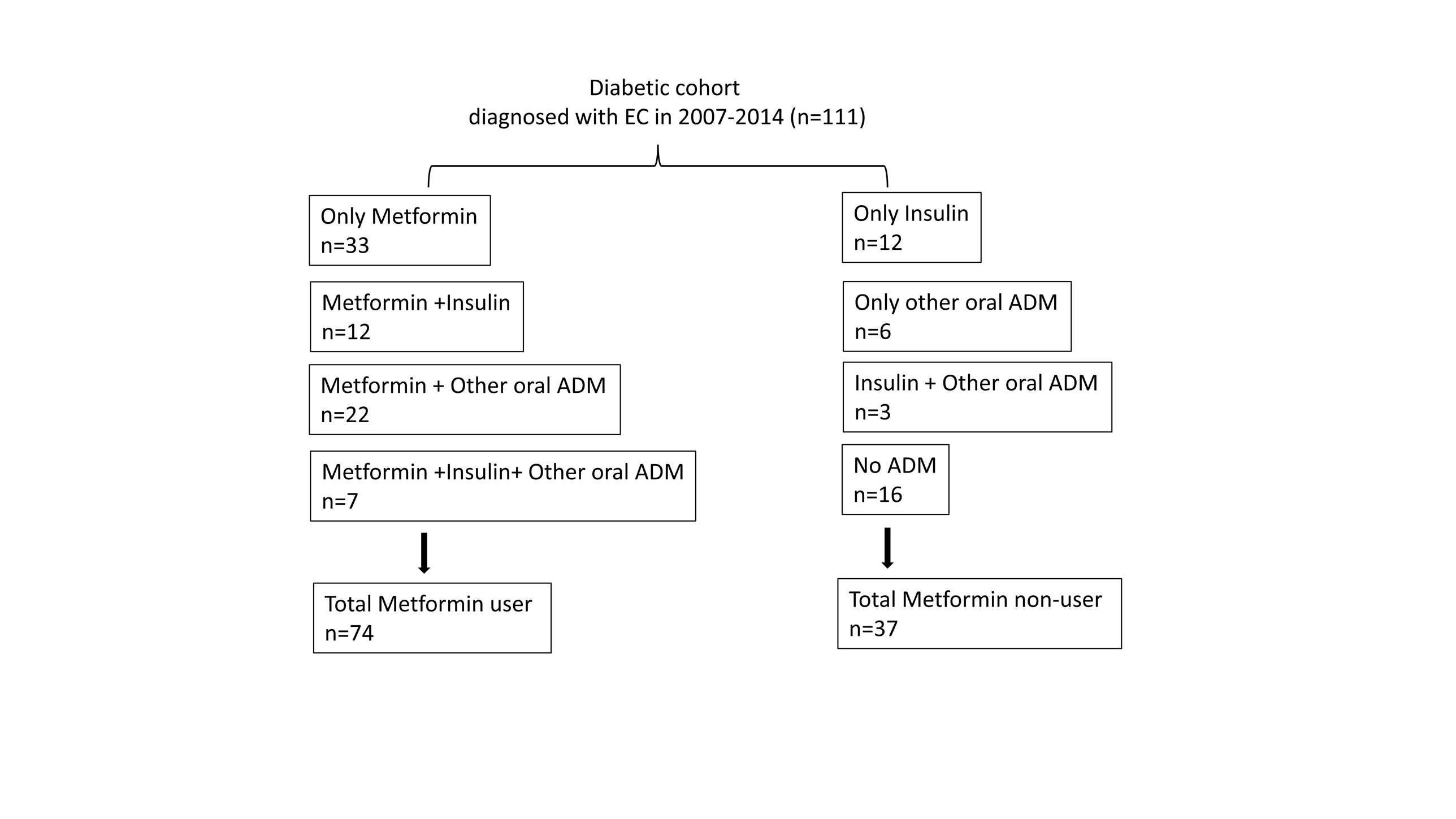

Supplement: Supplementary file 2 [file Image1.jpeg]

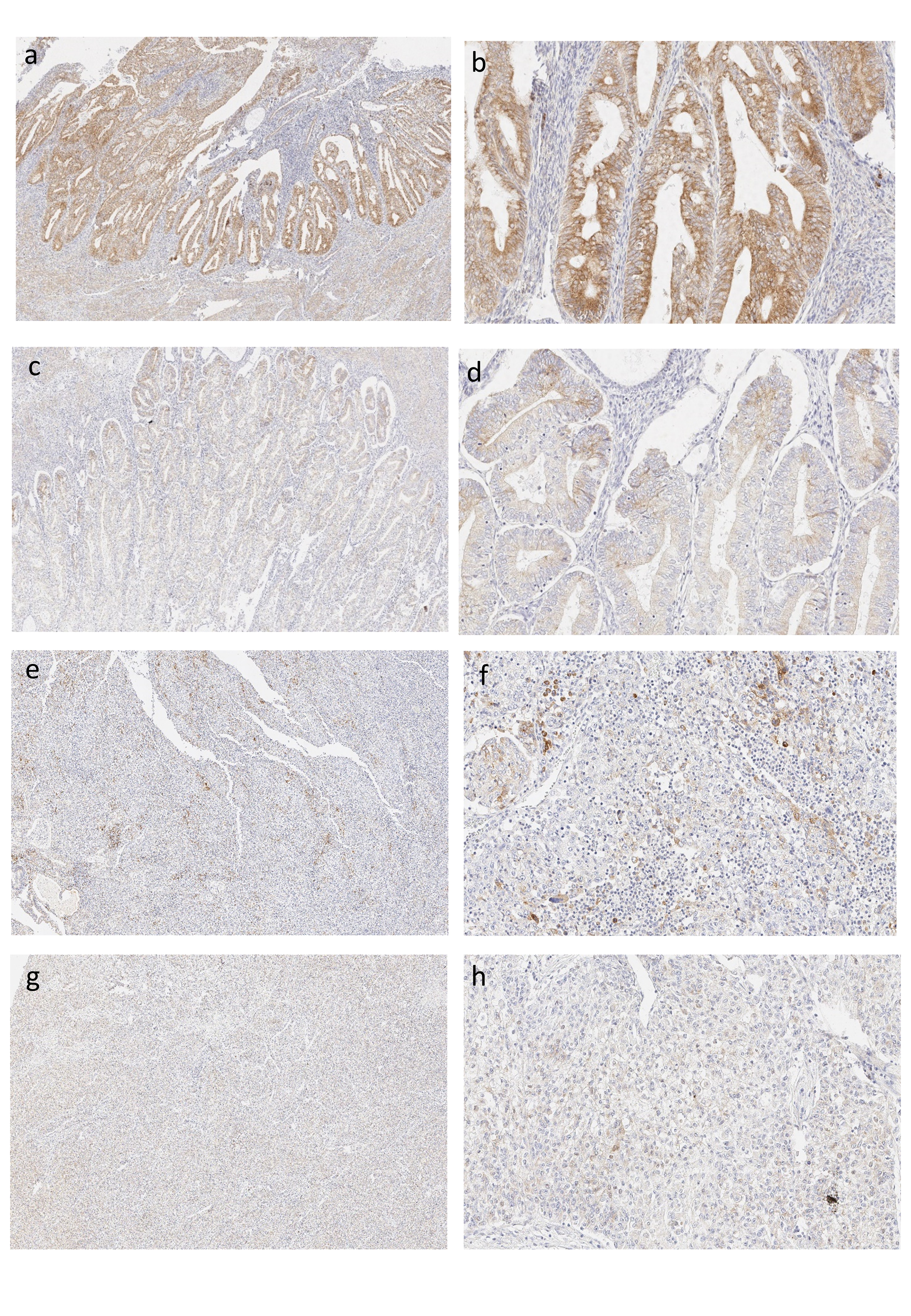

Supplement: Supplementary file 3 [file Image2.jpeg]
