# Supplementary material for: Low Expression of Stanniocalcin 1 (STC-1) Protein Is Associated With Poor Clinicopathologic Features of Endometrial Cancer
Source: Pathol Oncol Res. 2021 Sep 28;27:1609936. doi: 10.3389/pore.2021.1609936 (PMC8505533; doi:10.3389/pore.2021.1609936)
Supplement: Supplementary file 4 [file Table2.docx]

**Supplemental Table 2: Comparison of variables of epithelial STC-1 expression in EC patients from the diabetic cohort**

| **Risk variables** | **Weak expression**  **(Score 0-2)** | **Strong expression**  **(Score 3)** | **P-value** |
| --- | --- | --- | --- |
| Age >65 years | 52/74 (70.3%) | 24/37 (64.9%) | 0.563 a |
| Body mass index (BMI) ≥30 kg/m^2^ | 53/66 (80.3%) | 29/35 (82.9%) | 0.755 a |
| Histology Type 2 | 19/74 (25.7%) | 9/37 (24.3%) | 0.877 a |
| Stage Advanced (≥II) | 19/72 (26.4%) | 10/35 (28.6%) | 0.812 a |
| Myometrial invasion >50% | 28/70 (40.0%) | 11/30 (36.7%) | 0.754 a |
| Lymphovascular space invasion | 15/26 (57.7%) | 5/10 (50.0%) | 0.722 b |
| Cervical stromal invasion | 14/70 (20.0%) | 5/29 (17.2%) | 0.751 a |
| Positive peritoneal cytology (grade V) | 2/62 (3.2%) | 2/29 (6.9%) | 0.590 b |
| Tumor size >2 cm | 23/26 (88.5%) | 9/9 (100%) | 0.553 b |
| Metformin user | 50/74 (67.6%) | 24/37 (64.9%) | 0.776 a |

a Chi-Square test; b Fisher´s Exact Test (due to small number of cases in cells)
