# Supplementary material for: Low Expression of Stanniocalcin 1 (STC-1) Protein Is Associated With Poor Clinicopathologic Features of Endometrial Cancer
Source: Pathol Oncol Res. 2021 Sep 28;27:1609936. doi: 10.3389/pore.2021.1609936 (PMC8505533; doi:10.3389/pore.2021.1609936)
Supplement: Supplementary file 5 [file Table3.docx]

**Supplementary Table 3: Comparison of variables of metformin user *vs.* non -users in EC patients from the diabetic cohort.**

| **Risk variables** | **Metformin**  **(n = 74)** | **No metformin**  **(n = 37)** | **P-value** |
| --- | --- | --- | --- |
| Age >65 years | 48/74 (64.9%) | 28/37 (75.7%) | 0.248 a |
| Body mass index (BMI) ≥30 kg/m^2^ | 56/69 (81.2%) | 26/32 (81.3%) | 0.991 a |
| Histology Type 2 | 21/74 (28.4%) | 7/37 (18.9%) | 0.279 a |
| **Stage Advanced (≥II)** | **25/72 (34.7%)** | **4/35 (11.4%)** | **0.011 a** |
| **Myometrial invasion >50%** | **31/68 (45.6%)** | **8/32 (25.0%)** | **0.049 a** |
| Lymphovascular space invasion | 14/26 (53.8%) | 6/10 (60.0%) | 1.0 b |
| **Cervical stromal invasion** | **17/67 (25.4%)** | **2/32 (6.3%)** | **0.024 a** |
| Positive peritoneal cytology (grade V) | 3/61 (4.9%) | 1/30 (3.3%) | 1.0 b |
| Tumor size >2 cm | 20/23 (87.0%) | 12/12 (100%) | 0.536 b |
| Stromal STC-1 (score 2) (none was score 3) | 0/74 (0%) | 1/37 (2.7%) | 0.333 b |
| Epithelial STC-1 (score 3) | 24/74 (32.4%) | 13/37 (35.1%) | 0.776 a |

a Chi-Square test; b Fisher´s Exact Test (due to small number of cases in cells); Bold values indicates statistically significant (P ≤ 0.05)
